# Composition and decomposition of rhizoma peanut (*Arachis glabrata* Benth.) belowground biomass

**DOI:** 10.1038/s41598-022-14001-7

**Published:** 2022-06-15

**Authors:** Erick R. S. Santos, José C. B. Dubeux, Lynn E. Sollenberger, Michelle C. B. Siqueira, Flávia O. S. van Cleef, David M. Jaramillo, Luana Q. S. D. Zagato, Luana M. D. Queiroz, Liza Garcia, Carlos C. V. Garcia, Martin Ruiz-Moreno

**Affiliations:** 1 Department of Agricultural, Food, and Nutritional Science, University of Alberta, Edmonton, Canada; 2 North Florida Research and Education Center, University of Florida, Gainesville, USA; 3 Agronomy Department, University of Florida, Gainesville, USA; 4 Departamento de Zootecnia, Universidade Federal Rural de Pernambuco, Recife, Brazil; 5 Center of Nuclear Energy in Agriculture, University of São Paulo, São Paulo, Brazil; 6 USDA-ARS, U.S. Dairy Forage Research Center, Madison, USA; 7 Aberdeen Research and Extension Center, University of Idaho, Moscow, USA

**Keywords:** Ecosystem services, Grassland ecology

## Abstract

Roots and rhizomes can play an important role in nutrient cycling, however, few studies have investigated how their decomposition pattern is affected by defoliation and time of the year. This 2-year study evaluated root-rhizome composition and decomposition of a warm-season rhizomatous perennial legume [rhizoma peanut (RP; *Arachis glabrata* Benth.)] under continuous stocking or when defoliated by clipping every 56 days. A 168-days incubation trial was performed to determine disappearance of biomass and N and changes in acid detergent fiber (ADF), acid detergent insoluble N (ADIN), and C:N ratio. Additionally, three 56-days incubations were performed each year to evaluate the disappearance coefficient (*B*_0_) and relative decay rate (*k*). There were no treatment differences in any response for the 168-days incubation. After 168 days, 21 and 60% of initial biomass and initial N remained, respectively. Relative decay rate for OM and N were 0.0088 and 0.0035 g g^−1^ day^−1^, respectively. Carbon-to-N ratio decreased from 29 at day 0 to 17 at day 168. Concentration of ADIN increased from 6.9 to 19.3 g kg^−1^, plateauing at day 79. The *B*_0_ and *k* for remaining OM and N were greater in late than early season and could be explained by greater N concentration and lesser C:N ratio. Rapid decomposition, difference in C:N ratio from day 0 to 168, and the increase in ADIN concentration during incubation indicate large amounts of root-rhizome-soluble C at initiation of incubation. These data indicate that RP root-rhizome turnover is more responsive to season than defoliation frequency.

## Introduction

Rhizoma peanut (*Arachis glabrata* Benth.) is a warm-season, rhizomatous, perennial legume adapted to sandy soils in the southern Gulf Coast region of the USA. Across six different RP entries, the average annual amount of biologically fixed N present in shoots was 200 kg ha^−1^^1^. When growing in association with companion grasses, legume N can be shared via plant litter, root exudates, or, when grazing animals are present, via animal excreta^2–4^. For example, aboveground litter of bahiagrass (*Paspalum notatum* Flüggé) and RP mixtures had superior litter quality compared with grass monocultures^5^, and increasing proportion of RP in mixtures with grasses was correlated with increasing litter N release^6^. Similarly, adding the legume calopo (*Calopogonium mucunoides* Desv.) into perennial grass systems increased net annual N mineralization over grass monocultures^7^.

While contributions of legumes to nutrient cycling through aboveground plant litter have been studied extensively, root and rhizome residues also play an important role in nutrient cycling^2^, but have received much less attention. Root or root-rhizome inputs are especially important in grasslands because of the proportion of photosynthate and biomass allocated belowground^8,9^.

Quantity of RP belowground biomass is affected by genotype and defoliation management^1,10–13^. Across 14 RP entries, Cooley et al.^13^ reported that root-rhizome mass decreased from 8.5 to 5.5 Mg OM ha^−1^ when clipping frequency increased from once to twice a year. Frequent close grazing decreased RP root-rhizome mass^10^. Composition and turnover of grassland belowground biomass can also be affected by management. Root turnover of elephantgrass (*Pennisetum purpureum* Schum.) increased with greater stocking rate in the absence of N fertilization, however, it decreased when 300 kg N ha^−1^ was applied^14^. Seasonal differences in mass and composition of RP roots and rhizomes have also been reported^11^, suggesting that time of the year may affect belowground litter chemical composition and decomposition dynamics. Therefore, investigating decomposition patterns during different seasons may improve our understanding of root-rhizome turnover and nutrient cycling throughout the year.

Based on the literature, we hypothesized that RP root-rhizome *B*_0_ and *k* will vary as a function of season and defoliation frequency. Understanding such dynamics are important in predicting root-rhizome turnover and estimating its contribution to grassland ecosystems. The objectives of this study were (1) to investigate how N concentration, C:N ratio, ADF, and ADIN concentration affect decomposition patterns in RP root-rhizome biomass, and (2) to investigate how disappearance coefficient and relative decay rate of RP root-rhizome biomass are affected by defoliation frequency and time of the year.

## Results

### Long-term study

**Figure 1:**
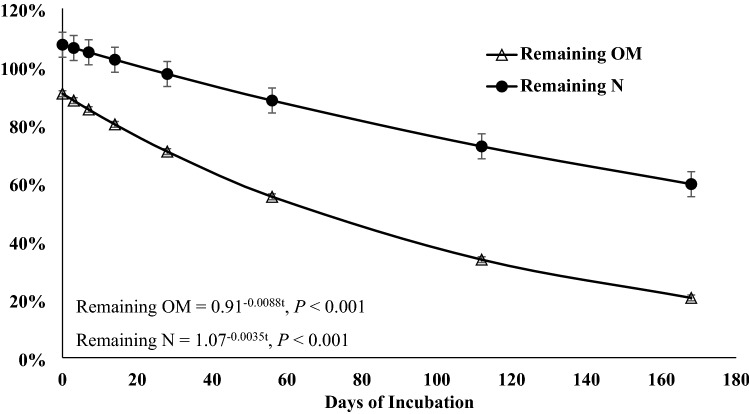
Remaining biomass (OM) and remaining N of rhizoma peanut roots and rhizomes during a 168-days incubation period. The values represent the averages of two treatments (grazed and non-grazed) and 2 years. Bars refer to the SE.

**Figure 2:**
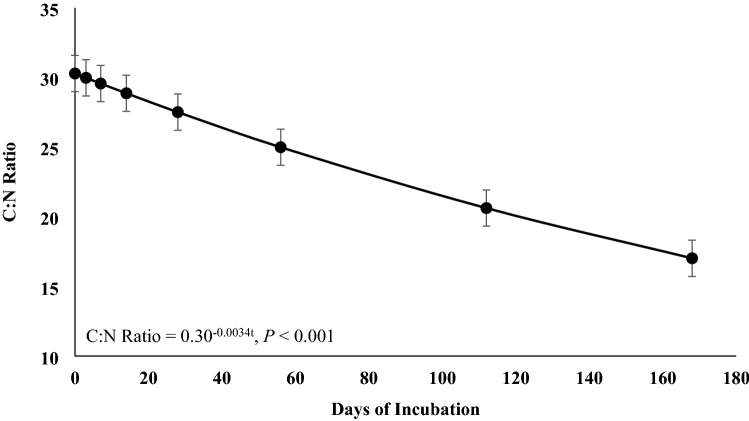
Carbon-to-nitrogen ratio of rhizoma peanut roots and rhizomes during a 168-days incubation period. The values represent the averages of two treatments (grazed and non-grazed) and 2 years. Bars refer to the SE.

**Figure 3:**
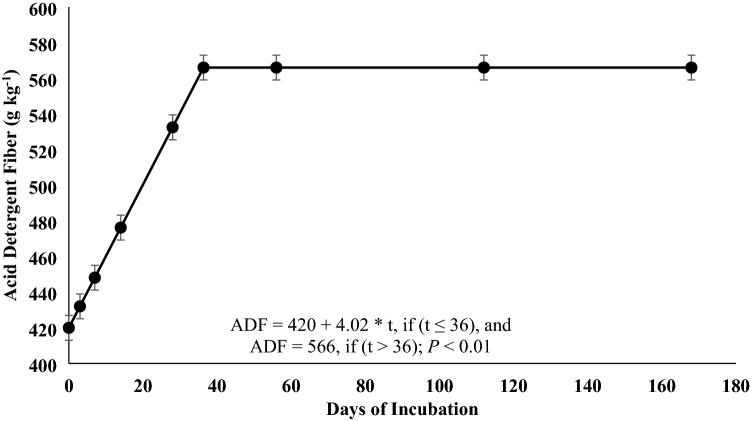
Acid detergent fiber of rhizoma peanut roots and rhizomes during a 168-days incubation period. The values represent the averages of two treatments (grazed and non-grazed) and 2 years. Bars refer to the SE.

**Figure 4:**
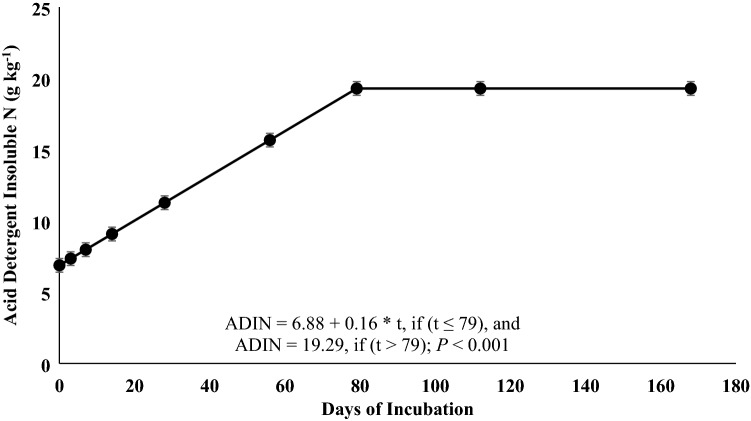
Acid detergent insoluble N of rhizoma peanut roots and rhizomes during a 168-days incubation period. The values represent the averages of two treatments (grazed and non-grazed) and 2 years. Bars refer to the SE.

There were no significant differences among treatments or for the interaction of treatment × day of incubation for any response variable (*P* > 0.05), but day of incubation was significant for all variables (*P* < 0.01). Remaining OM (Fig. 1), remaining N (Fig. 1), and C:N ratio (Fig. 2) responses were explained by the single negative exponential model. Only 21% of the original OM and 60% of the original N remained at the end of the incubation period. The relative decomposition rate (*k*) for OM and N were 0.0088 and 0.0035 g g^−1^ day^−1^. Carbon-to-N ratio decreased from 30 to 17 from day 0 to day 168, with a *k* of 0.0034 g g^−1^ day^−1^. Acid detergent fiber (Fig. 3) and ADIN (Fig. 4) concentrations fit the linear plateau model. Concentration of ADF increased from 420 at day 0 to 566 g kg^−1^, at day 36, when it reached the plateau. Concentration of ADIN increased from 6.9 at day 0 to 19.3 g kg^−1^, reaching the plateau at day 79.

### Short-term studies

There was a treatment × season interaction for initial N concentration (*P* < 0.001) and C:N ratio (*P* < 0.01). The N concentration prior to incubation was greater for grazed than non-grazed in the late season, whereas treatments did not differ in the other seasons (Table 1). Additionally, the grazed treatment had greater N concentration in the late season than during the other two seasons of the year. Conversely, N concentration in the non-grazed site was less during the late season than early and middle seasons.

**Table 1:** Treatment × season interaction on N concentration (*P* < 0.001; SE = 0.64) and C:N ratio (*P* < 0.01; SE = 1.93) of RP roots and rhizomes collected at three different times of the year.

Treatment	Season		
	Early	Middle	Late
**Nitrogen (g kg**^**−1**^**)**			
Grazed	12.2a^†^B^‡^	11.8aB	14.7aA
Non-Grazed	12.5aA	12.2aAB	11.1bB
**C:N ratio**			
Grazed	35.5aA	36.2aA	28.9bB
Non-Grazed	34.6a	35.3a	37.9a

^†^Different letters within the same column and same response are statistically different at the 5% probability level.

^‡^Different letters within the same row are statistically different at the 5% probability level. Early season: 2 May 2018 and 1 May 2019. Middle season: 27 June 2018 and 26 June 2019. Late season: 23 Aug. 2018 and 21 Aug. 2019.

Carbon-to-nitrogen ratio was less for root-rhizome biomass from the grazed than non-grazed treatment during the late season (28.9 vs. 37.9, respectively), nonetheless, treatments did not differ among each other during the other two seasons. Additionally, within the grazed treatment, the late season had a lesser C:N ratio value than the early (35.5) and middle (36.2) seasons.

The *B*_0_ and *k* for remaining OM and N were not affected by treatment (*P* > 0.05), nonetheless, they were affected by season (Table 2). The *B*_0_ for OM was lesser (*P* < 0.001) during the late season (0.90) than for the early (0.96) and middle (0.95) seasons. Organic matter *k* was greater (*P* < 0.05) during the late (0.0168 g g^−1^ day^−1^) than during the middle season (0.0117 g g^−1^ day^−1^), however, there was no difference between the early season (0.0142 g g^−1^ day^−1^) and the other two seasons.

**Table 2:** Disappearance coefficient (*B*_0_) and relative decay rate (*k*) for organic matter and nitrogen of rhizoma peanut roots and rhizomes incubated in situ for a period of 56-days, at three different times of the year.

Season	Organic matter (*B*_0_)	Organic matter (*k*)	Nitrogen (b0)	Nitrogen (*k*)
Early	0.96a^†^	0.0142ab	1.01a	0.0041b
Middle	0.95a	0.0117b	1.02b	0.0069a
Late	0.90b	0.0168a	0.95c	0.0060a
standard error	0.01	0.0041	0.03	0.0012
*P*-value	< 0.001	< 0.05	< 0.001	< 0.001

The values represent the averages of two treatments (grazed and non-grazed) and 2 years.

^†^Different letters within the same column are statistically different at the 5% probability level.

The *B*_0_ for N was lesser (*P* < 0.001) during the late season (0.95) than during the early (1.01) or middle (1.02) seasons, however, there was no difference between early and middle seasons. Relative decay rate for N increased from 0.0041 g g^−1^ day^−1^ in the early season to 0.0069 g g^−1^ day^−1^ in the middle season (*P* < 0.001). In the late season, N relative decay rate was 0.0060 g g^−1^ day^−1^, and it did not differ significantly from the middle season.

## Discussion

Differences among treatments probably did not occur in the long-term decomposition study because impact of the grazing treatments on belowground structures was likely limited early in the growing season when sampling occurred and also because root-rhizome biomass and composition of Ecoturf RP is less responsive to defoliation management than some RP cultivars and germplasms^15^. In a simultaneous study, Santos et al.^16^ reported no significant difference for the root-rhizome N pool for the grazed and non-grazed treatments for this trial. Root-rhizome N pool at the beginning of the study was 180 kg N ha^−1^. Considering that there was 60% of remaining N (40% disappearance) at the end of the incubation period (168 days), we could estimate that in a pure stand of RP roots and rhizomes can provide a total of 72 kg N ha^−1^ during this time, or 0.43 kg N ha^−1^ day^−1^. The accuracy of this estimate depends upon actual turnover rates of root and rhizome which were not quantified in this study.

The rapid biomass disappearance from roots and rhizomes can be associated with the presence of soluble C at the beginning of the incubation period. The C:N ratio decreases over time because soluble C decomposes rapidly, whereas the N bound to the fiber slows the rate of N losses^5,17–19^. The proportion of N bound to the acid detergent fiber increased from 6.9 at day 0 to 19.3 g kg^−1^ at day 79, when it reached a plateau, concurring with the decrease in the C:N ratio during the incubation period. Systems with RP can improve N cycling by increasing litter quality and decomposition rate^5,6^. Additionally, soil under RP had greater particulate organic N than fields dominated by perennial weeds^20^, and the addition of legumes into warm-season grass monoculture systems may increase N mineralization rate^7^.

The initial value for RP root and rhizome N concentration and C:N ratio were in the range of values previously reported in the literature^1,11,12,21^. In a 2-year study, when RP was harvested three or four times a year, Santos et al.^12^ reported N concentration and C:N ratio for Ecoturf RP belowground material of 14.0 and 30.4 g kg^−1^, respectively. Mullenix et al.^11^ evaluated the belowground responses of four RP entries, including Ecoturf, under two defoliation frequencies (3 or 6 week) and two grazing intensities (50 or 75% of herbage removal). They did not report any differences among the treatments applied, however, during the first year of study, the authors reported a date × entry interaction for N concentration, with a decrease from 18 in June to 15 g kg^−1^ in October. The RP root-rhizome N concentration in our study was affected by the treatment × season interaction. Compared to the early season, the N concentration for the non-grazed treatment was significantly less, whereas the RP root-rhizome N concentration for the grazed site increased. Such response was probably caused due to the forage mass at the non-grazed site being consistently greater than at the grazed site, resulting in greater N allocation to the aboveground material, whereas the grazed site increased the N concentration in the belowground material towards the end of the year, probably as a phenotypic plasticity response to grazing pressure (Shepard et al.^15^). In a simultaneous study, Santos et al.^16^ reported that total harvested N (61 kg N ha^−1^) in the aboveground material was greatest at the time that the belowground material for the late season decomposition trial was collected, compared to the other seasons.

The low disappearance coefficient and faster relative decay rate for OM and N during the late season indicate a more accelerated decomposition rate during this time of the year. Despite differences among treatments not being reported for these parameters, the material utilized for the incubation during the late season from the grazed treatment had greater N concentration (14.7 g kg^−1^) and lesser C:N ratio (Table 1), which was probably the cause for a more rapid disappearance during this time of the year. To our knowledge, there are no studies evaluating RP root and rhizome decomposition dynamics. Additionally, litter decomposition studies usually are performed using only one incubation period per year^5,6,14,17^. As the proportion of RP increases, the aboveground litter decomposition of bahiagrass-RP increases, because of the improvement in litter quality^5,6^. Silva et al.^7^ indicated that N net mineralization in aboveground litter is enhanced when legumes are added into warm-season grass monocultures. However, our results indicate that such decomposition parameters may not follow the same pattern throughout the year, especially in a grazing system. As the proportion of nutrients return via aboveground litter and excreta vary in a grazed vs. non-grazed system^2^, similar responses may occur with the belowground biomass, since root systems are affected by grazing management^22^. Silver and Miya^23^ indicated that the largest amount of variability in root decay could be explained by Ca and C:N ratios, with a smaller proportion explained by latitude, mean annual temperature, mean annual precipitation, and actual evapotranspiration. In addition, in a stepwise multiple linear regression, the authors indicated that actual evapotranspiration, root Ca, and C:N ratio accounted for approximately 90% of the variability in root decay. Because wheatear condition varies throughout the year, such parameters may have played an important role on root-rhizome decay.

## Summary and conclusions

Roots and rhizomes biomass and N disappearance were not affected by exclusion from grazing when incubated for 168-days. The proportion of N bound to the acid detergent fiber increased along the 168-days period, reaching the plateau at day 79. Conversely, the C:N ratio decreased along the incubation period, which indicated high contents of soluble C at the beginning of the incubation. The 56-days incubation trial showed that the disappearance coefficient and relative decay rate are affected by season of the year and they can be explained by factors such as the initial N concentration and C:N ratio. Rhizoma peanut roots and rhizomes play an important role on N cycle and this contribution is often overlooked due to the biological N fixation be primarily reported for the aboveground portion of the plant. Understating the dynamics of these components is an important tool to take into consideration when designing RP-grass based grazing systems.

## Materials and methods

### Experimental site

All procedures for the experiment involving animals were carried out in accordance with relevant guidelines and regulations and they were approved by the Institutional Animal Care and Use Committee (IACUC) of the University of Florida (protocol #201509019). The experiment was conducted at the University of Florida North Florida Research and Education Center (NFREC) located in Marianna, FL (30° 52ʹ N, 85° 11ʹ W, 35 m asl) during 2018 and 2019.

The study site was an existing mixed RP-bahiagrass grazing study where ‘Ecoturf’ RP was strip-planted into ‘Argentine’ bahiagrass on 12 June 2014. Rhizoma peanut strips were approximately 2-m wide, making it possible to harvest RP forage, roots, and rhizomes free of bahiagrass contamination^3,4^. The RP was collected from a nursery at the University of Florida—NFREC, whereas the bahiagrass seeds were bought from a seed company. All plants were collected, purchased, managed, and the research was conducted in compliance with relevant institutional, the corresponding national, and international guidelines and legislation.

**Figure 5:**
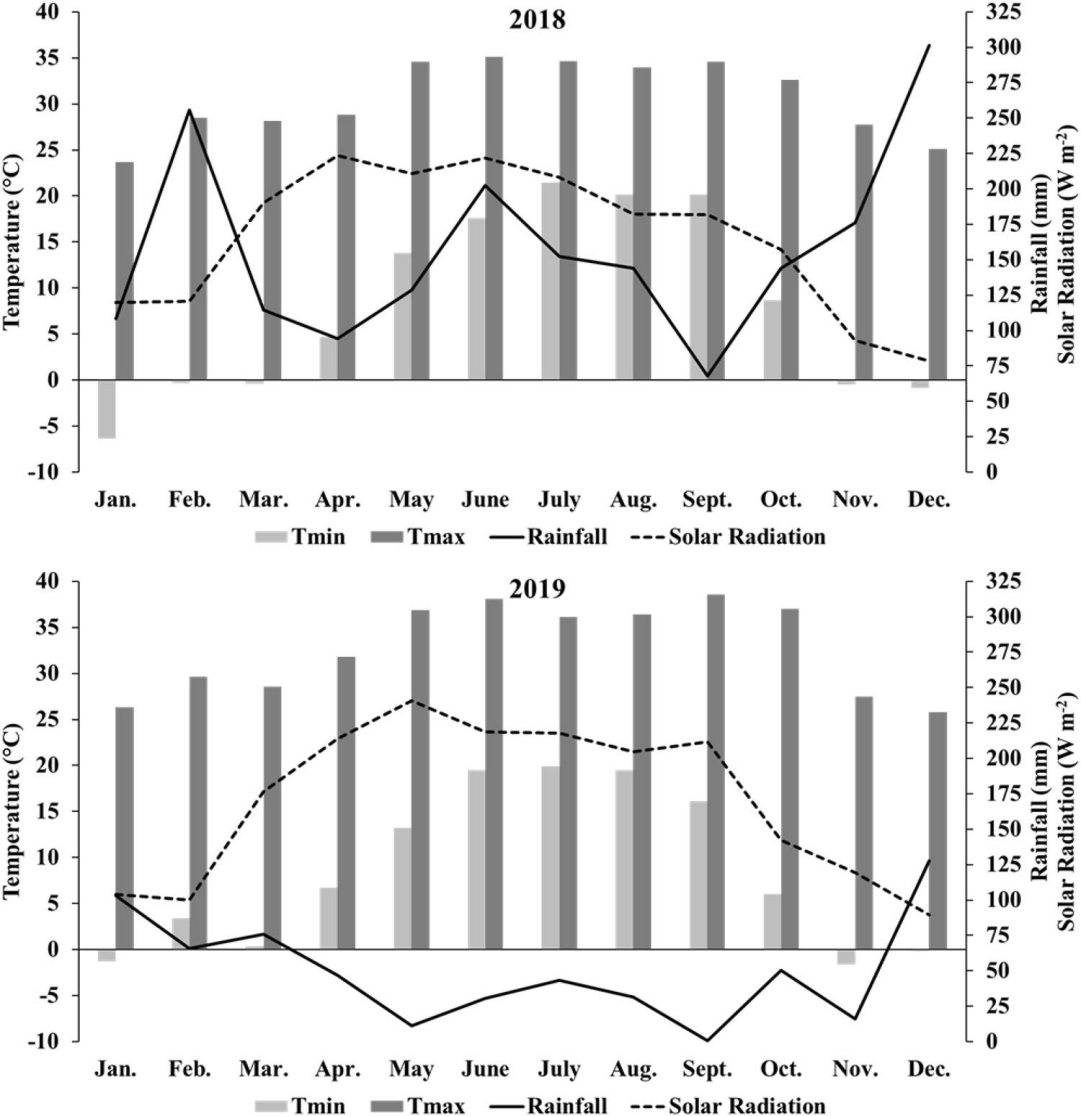
Monthly weather conditions at North Florida Research and Education Center (NFREC) Marianna, FL, during the experimental years.

Soils at the experimental site were classified as Orangeburg loamy sand (fine-loamy, kaolinitic, thermic Typic Kandiudults^24^. At the beginning of the study, soil pH was 5.7 and soil OM was 15.4 g kg^−1^. Additionally, Mehlich-I extractable soil P, K, Mg, and Ca concentrations at the beginning of the experiment were 26, 99, 43, and 224 mg kg^−1^, respectively. Total annual rainfall and average annual temperature at the experimental site were 1889 and 602 mm, and 19 and 21 °C, for 2018 and 2019, respectively, and their monthly averages are shown in Fig. 5.

### Treatments and experimental design

Treatments were two defoliation regimes applied to RP, continuously stocking and 56-days interval between clipping harvests. At the continuous stocking, stocking rates were variable to maintain similar herbage allowance among pastures, which was assessed every 14 days as described by Sollenberger et al.^25^. Two tester Angus crossbred steers (*Bos* spp.) remained on each pasture throughout the experimental period. Put-and-take cattle were allocated as needed to maintain a target herbage allowance of 1.5 kg DM kg^−1^ bodyweight^3^. Treatments were situated adjacent to each other (i.e., paired sites) in monoculture strips of RP within each of three 0.85-ha pastures. Each pasture was considered a block, thus the experiment consisted of three replicates of each treatment in a randomized complete block design. Within each replicate, treatments had three repetitions (pseudo replicates). To prohibit animal access to the non-grazed treatment, three 2 × 2-m exclusion cages were placed on RP strips in each pasture. Rhizoma peanut herbage mass was determined at both the grazed and non-grazed sites three times each year, at days 56, 112, and 168 of the experimental period by using a 0.25-m^−2^ quadrat. Two quadrats were collected in each repetition by clipping all the biomass within each quadrat at 2-cm stubble height. After each herbage mass sampling, the non-grazed residual dry matter inside the cages was clipped to a 2-cm stubble height using a weed eater and the herbage removed by raking. On average, across sampling dates and years, herbage mass at the grazed and non-grazed sites was 1050 and 1810 kg of organic matter (OM) ha^−1^, respectively.

### Long-term and short-term decomposition studies

There were two types of root-rhizome decomposition trials. The first is referred to as the *long-term* decomposition study, and the second is the *short-term* decomposition study. The long-term study had an incubation period of 168 days, with a single in-situ incubation per year starting in May. The short-term study had in-situ incubation periods of 56 days and there were three incubations per year, occurring in May, June, and August. In all cases, only roots and rhizomes attached to the plant were used in both trials.

#### Long-term study

On 26 Apr. 2018 and on 23 Apr. 2019, right after RP emergence after breaking dormancy, RP roots and rhizomes were collected from an existing mixed RP-bahiagrass grazing study where RP had been planted in strips into bahiagrass (*Paspalum notatum* Flüggé) in 2014. Rhizoma peanut strips were approximately 2.75-m wide, alternating with similar wide bahiagrass strips. A pure stand of RP had been maintained in the strips during previous years using herbicides^3^, making it possible to harvest RP roots and rhizomes free of bahiagrass contamination. Roots and rhizomes were collected at 24 different points in each of three blocks of the original experiment. Roots and rhizomes were collected at 20-cm depth using shovels. As defoliation treatments had not being applied at this time of the year, the same material was used to perform the incubation inside and outside the exclusion cages. After harvesting, excess soil was removed by shaking from the root-rhizome mat using a 1.4-cm diameter sieve. Thereafter, the existing aboveground material was clipped, and the roots and rhizomes were then washed over the same sieve to remove the remaining soil. After washing, roots and rhizomes were dried to constant weight in a forced-air drying oven at 55 °C.

To perform the decomposition study, approximately 12 g of dry roots and rhizomes were placed in Ankom bags (10 by 20 cm, 50 µm porosity; ANKOM Technology) and sealed^17^. Roots and rhizomes were aimed to be placed intact into Ankom bags, nonetheless, when they could not fit inside the bags, they were cut in the middle before being placed. On 2 May 2018 and 1 May 2019, the incubation period began. For each treatment, bags were incubated in situ in the field at 10-cm depth in the same blocks from which they were collected. Bags were removed from the field after 0, 3, 7, 14, 28, 56, 112, and 168 days. For each treatment within each block, three bags were incubated for each incubation time. Additionally, empty bags (one bag per treatment per time per block) were placed in the field. After removal of the in-situ bags from the field, samples and empty bags were dried at 55 °C for 72 h, cleaned with a brush, and weighed. Thereafter, samples were ground to pass a 2-mm screen using a Wiley Mill (Model 4, Thomas-Wiley Laboratory Mill, Thomas Scientific) and analyzed for DM and OM. Subsamples of the 2-mm ground samples were ball milled in a Mixer Mill (MM 400, Retsch) at 25 Hz for 9 min. Ball-milled samples were analyzed for C and N by dry combustion using an elemental analyzer (Vario Micro cube, Elementar). Additionally, samples ground at 2-mm were used to determine ADF in aboveground samples^26^. The N concentration in the ADF was determined using the above protocol to obtain the ADIN.

#### Short-term studies

The short-term studies were performed following the same procedures as the long-term study, except that the incubation period was only 56 days, and these studies were repeated three times each year. Roots and rhizomes were incubated in situ on 2 May, 27 June, and 23 Aug. 2018 and on 1 May, 26 June, and 21 Aug. 2019, following the same protocol as described above, except that bags were removed from the field after 0, 3, 7, 14, 28, and 56 days of incubation. The incubations occurring in May, June, and August will be referred as early, middle, and late season, respectively.

The early-season incubation period uses the data from the first 56 days of the long-term study described above. For the middle- and late-season incubations each year, roots and rhizomes were harvested approximately 7 days days prior to incubation. Approximately six points in each repetition were collected at 20-cm depth using shovels. For the grazed treatment, roots and rhizomes were collected in the grazed area nearby the exclusion cages, whereas for the non-grazed treatment, the material was collected inside the exclusion cages. After removal of the bags from the field, they were processed and analyzed for DM, OM, C, and N following the protocol described above.

### Statistical analyses

#### Long-term study

Remaining biomass, remaining N, C:N ratio, ADF, and ADIN were analyzed using the PROC GLIMMIX from SAS^27^, with treatment and days of incubation as fixed effects, and years and blocks as random effects. Days of incubation were considered repeated measures. Means were compared using the PDIFF procedure at the 5% significance level. When treatment or the interaction of treatment × day of incubation were statistically significant in the ANOVA, nonlinear models were tested to fit the data for each variable and treatment. Nonlinear models were selected for a given response based on data distribution and type of response. If only days of incubation was significant, the same model was applied for all treatments.

Remaining biomass (OM basis), remaining N, and C:N ratio were explained by the single exponential decay model^14,17,28^. The equation describing this process is:

1 $$X=B0\, {exp}^{-kt},$$

where *X* is the remaining biomass, remaining N, or C:N ratio at day *t*, *B*_*0*_ is the disappearance coefficient, and *k* is the relative decay rate (g g^−1^ day^−1^). The model used to describe ADF and ADIN was the two-stage model “linear plateau”^15,29^. The equation describing this process is:

2 $$\begin{gathered} Xt = A + b1 \times t\, {\text{if t }} \le {\text{ T}}, \hfill \\ {\text{and}},{ } Xt = A + b1 \times T\, {\text{if t }} > {\text{ T}}, \hfill \\ \end{gathered}$$

where *X* is the concentration of ADIN, *t* is the day of incubation, *A* is the initial concentration, *b1* is the rate of increase in concentration from the beginning of incubation until plateau is reached; and T is the day in which concentration reaches the plateau.

#### Short-term studies

The single exponential model was applied in the remaining OM and remaining N, for each experimental unit, to obtain individual values for *B*_0_ and *k*. The data for initial N concentration, initial C:N ratio, and *B*_*0*_ and *k* for remaining OM and remaining N were analyzed using the PROC GLIMMIX from SAS^27^, with treatment and period as fixed effects, and years and blocks as random effects. Means were compared using the PDIFF procedure at the 5% significance level.

### Arrive guidelines

This is study is reported in accordance to ARRIVE guidelines.

## Data Availability

The datasets used and/or analyzed during the current study available from the corresponding author on reasonable request.

## Abbreviations

ADF: Acid detergent fiber
ADIN: Acid detergent insoluble N
OM: Organic matter
RP: Rhizoma peanut
